# The Effect of Baseline Patterns of Spiritual Coping, Forgiveness, and Gratitude on the Completion of an Alcohol Addiction Treatment Program

**DOI:** 10.1007/s10943-021-01188-8

**Published:** 2021-01-30

**Authors:** Edyta Charzyńska

**Affiliations:** Faculty of Social Sciences, University of Silesia in Katowice, ul. Grażyńskiego 53, 40-126 Katowice, Poland

**Keywords:** Alcohol dependence, Spirituality, Religious coping, Forgiveness, Gratitude

## Abstract

The purpose of this study was to identify distinct profiles of persons beginning alcohol addiction therapy with similar baseline configurations of spiritual coping, forgiveness, and gratitude. The associations between latent profile membership and the completion of therapy were also examined. The sample was composed of 358 alcohol-dependent persons receiving an outpatient treatment program. The Spiritual Coping Questionnaire, the Forgiveness Scale, and the Gratitude Questionnaire were used to assess the baseline levels of spirituality-related variables. Using latent profile analysis, five profiles were identified: (1) both moderately positive and negative dimensions of spirituality (33.2%), (2) moderately positive dimensions of spirituality (21.0%), (3) predominantly negative dimensions of spirituality (20.2%), (4) mixed dimensions of spirituality with the lowest positive religious coping (14.0%), and (5) highly positive dimensions of spirituality (11.6%). Notably, the latent profiles differed in terms of the treatment completion rates. The results suggest the need to carry out a multidimensional assessment of spiritual functioning of persons beginning alcohol addiction therapy to provide treatment that is adjusted to patients’ spiritual potential and deficits.

## Introduction

Spirituality is a concept that has been connected with alcohol dependence for many years (Vaillant 1983). The cultivation of spirituality is a hallmark of Alcoholics Anonymous (AA 1972). Members of AA believe that drinking problems stem from the experiences of inner emptiness, the lack of purpose and meaning of life, and the destruction of moral virtues, especially from being resentful, unable to forgive, and self-centeredness (AA 1972, 2001; Twerski 1997). The AA 12-step program of recovery involves believing in, accepting, and relying on a power higher than self, changing personal values, and helping other persons with alcohol dependence by taking the message of spiritual awakening to them (AA 2001; Miller 1998). The spiritual philosophy of AA has been implemented in numerous formal alcohol treatment models, especially the Minnesota model, which assumes that alcohol dependence affects persons physically, mentally, socially, and spiritually, and thus, each dimension of functioning should be included in therapy to provide adequate care (McCrady et al. 2014).

For the past two decades, addiction researchers also have exhibited a rising interest in spirituality and its manifestations (Cook 2004; Jarusiewicz 2000; Miller 1998). The current study is intended to advance previous work on spiritual coping and spirituality-related virtues—forgiveness and gratitude—and on the role they play in alcohol addiction therapy.

## Definitions

### Spiritual Coping

In this study, the widely accepted concept of spirituality and religiousness as overlapping yet distinct and distinguishable constructs has been applied (Burkhardt and Solari-Twadell 2001; Jarusiewicz 2000). According to Wong (2010), spirituality grants the individual the ability to recognize the resourcefulness of meaningful living, in this way providing the opportunity to use spirituality to transcend the detrimental effects of stressful situations. In a similar vein, Pargament et al. (2000) posit that during times of acute stress people often mobilize spiritual resources to mitigate the consequences of stressful circumstances. Analogously to the definition of religious coping (Pargament 1997), spiritual coping can be defined as the utilization of spirituality as a strategy for managing stressful life events. Such a definition of spiritual coping is not confined exclusively to religious coping, but it also includes other domains in which spirituality may be manifested—especially personal, social, and environmental ones (Burkhardt and Solari-Twadell 2001).

Conceptualizations of spiritual coping distinguish two forms: positive spiritual coping that is usually salubrious for an individual, and negative spiritual coping that is usually detrimental to health and well-being (Ano and Vasconcelles 2005; Pargament 1997). Moreover, the two forms of spiritual coping are relatively independent from each other, that is, when struggling with a certain situation, a person may resort to both of them (Charzyńska 2015; Pargament 1997).

Charzyńska (2015) proposed the conception of multidimensional spiritual coping and presented its operationalization, the Spiritual Coping Questionnaire (SCQ). SCQ encompasses four forms of positive spiritual coping: (1) personal (looking for inner peace, harmony, and serenity); (2) social (establishing deep and valuable relationships with other people); (3) environmental (searching for tranquility and peace in nature); and (4) religious (seeking support and strength from a stable relationship with God or a higher being). It also includes three forms of negative spiritual coping: (1) personal (questioning the meaning and purpose of one’s life); (2) social (focusing on the belief that people are egoistic and hypocritical); and (3) religious (questioning God’s or a higher being’s love and care for humans).

### Forgiveness and Gratitude

Forgiveness is one of the fundamental premises of world religions, spiritual formations, and moral systems (Webb et al. 2006; Worthington et al. 2001). Most researchers support the idea that various aspects of forgiveness can be distinguished on the basis of the method of forgiveness (e.g., offering, seeking, and feeling) and its target (e.g., self, others, deity, community, families, the universe; Toussaint and Webb 2005). The aspects of forgiveness most often studied in alcohol research involve self-forgiveness, forgiveness of others, and feeling forgiven by God. Self-forgiveness is defined as letting go of negative emotions (such as shame, guilt, or resentment) targeted at self, which are evoked by committing an act perceived by a person as morally wrong, and replacing them with more benevolent beliefs, emotions, and behaviors toward self (Hall and Fincham 2005). Forgiveness of others is understood as an intentional process involving affective, behavioral, and cognitive components, that entails the reduction of negative responses to an offender and in some cases also encompasses attempts to develop more positive and prosocial attitude toward him or her (Enright and Fitzgibbons 2015; Worthington et al. 2001). The other aspect of forgiveness—feeling forgiven by God—involves the recognition that God/a higher being has forgiven faults and other behaviors that are perceived by the individual as morally wrong (Toussaint et al. 2001).

Gratitude is another moral virtue highly related to religion and spirituality (Peterson and Seligman 2004). It is understood as a life orientation toward perceiving and appreciating good things in one’s life and the positive aspects of the world, even in difficult circumstances (Wood et al. 2008). Gratitude is often seen as being conceptually related to forgiveness (Breen et al. 2010). Indeed, both are prosocial and empathy-based virtues that enhance well-being and health by reinforcing the establishment and maintenance of long-lasting positive relationships with self, others, and God (McCullough et al. 2002; Wood et al. 2008).

## Research on Alcohol Dependence and Spiritual Functioning

A substantial number of studies have demonstrated that spirituality is a protective factor against alcohol dependence (Giordano et al. 2015; Miller 2013). It has also been noted that extensive drinking has a negative influence on spiritual involvement and spiritual well-being (Miller 1998). Moreover, spirituality is known to support recovery from alcohol dependence by providing individuals with a sense of purpose, meaning in life, and optimism, buffering stress and increasing social support (Jarusiewicz 2000; Lyons et al. 2010; Miller 1998,2013; Pardini et al. 2000; Piderman et al. 2008). Alcohol addiction therapy may also evoke substantial improvements in the spirituality domain, in some cases leading to the experience of spiritual awakening (Strobbe et al. 2013).

Surprisingly, despite these well-documented associations between spirituality and alcohol dependence as well as between stress and drinking problems (e.g., the stress-coping model of addiction; Wills and Hirky 1996), few studies have explored the impact of spiritual coping on alcohol dependence and its treatment. Further, all of them included only religious coping, excluding other domains of spiritual coping. For instance, Medlock et al. (2017) conducted a study among 331 persons with substance abuse disorders who were admitted to a psychiatric hospital for inpatient detoxification. In this study, positive religious coping correlated with greater mutual-help participation, fewer days of substance use in the previous month, and lower substance craving during detoxification. Conversely, negative religious coping was associated with higher drug craving and lower confidence in the ability to maintain abstinence post-discharge. In another study (Robinson et al. 2011), a six-month drop in negative religious coping among 364 alcohol-dependent individuals predicted more favorable drinking outcomes at nine-month follow-up. Overall, the studies suggest that positive religious coping may enhance outcomes of alcohol addiction therapy, while negative religious coping may be a barrier to treatment success (Puffer et al. 2012).

Analogously to religious coping, forgiveness is also known to play an important role in treatment of alcohol dependence. Statistically, resentment, hostility, and anger are higher in alcohol- and other substance-dependent people than in other populations (Lin et al. 2004). Excessive drinking of alcohol is often seen as a maladaptive strategy of coping with resentment or shame (Morrison et al. 2012). Moreover, anger was found to be a common risk factor for relapse (Levy 2008). Thus, as an adaptive coping strategy that helps to mitigate negative emotions toward others and toward self, and to improve self-esteem (Lin et al. 2004), forgiveness is recognized to constitute recovery capital for alcohol-dependent persons (Laudet and White 2008). Indeed, in the aforementioned study by Robinson et al. (2011), six-month changes in a general measure of forgiveness predicted improvements in nine-month drinking outcomes. Notably, a six-month increase in forgiveness of self had a stronger effect on nine-month favorable drinking outcomes than forgiveness of others. In another study Webb et al. (2006) examined the relationships between aspects of forgiveness (namely forgiveness of self, of others, and by God), alcohol use, and alcohol-related consequences among 157 people entering alcohol addiction treatment. At baseline, all aspects of forgiveness were inversely correlated with alcohol use and drinking consequences variables. However, relationships differed depending on the aspect of forgiveness and kind of alcohol-related variables. At follow-up, only forgiveness of self was associated with fewer drinking consequences. Further, baseline forgiveness of self and forgiveness of others were correlated with fewer drinking consequences at six-month follow-up. These studies suggest the importance of including different aspects of forgiveness in alcohol research and the need to explore their unique impact on drinking-related outcomes among alcohol-dependent persons.

With regard to the role of gratitude in alcohol addiction and its treatment, the data are scarce. Those available demonstrate the salutary effect of gratitude on recovery through several mechanisms: giving meaning to life, developing and maintaining beneficial social relationships, providing more adaptive coping strategies, and improving quality of life (Chen 2017; Nelson 2009). A study carried out by LaBelle and Edelstein (2018) among members of the 12-step addiction recovery program (*N* = 184) showed that gratitude was associated with better indicators of recovery, that is, the 12-step practices and AA promises, and better general life outcomes (i.e., fewer stress and health symptoms; more post-traumatic growth and social support). Similarly, in another study (Krentzman et al. 2015), participating in a 14-day web-based gratitude intervention turned out to catalyze positive cognitions and reinforce recovery among persons attending outpatient treatment for alcohol dependence.

## The Current Study

Although spirituality is a multidimensional construct that includes many types of specific beliefs and behaviors (Cook 2004; Miller 1998), much research has focused on spirituality in a broad, general sense rather than on its specific dimensions. Even in those studies that treated spirituality multidimensionally, manifestations of spirituality and spirituality-related characteristics were usually analyzed separately, without considering their different combinations. This topic was brought up by Barton and Miller (2015) in a study conducted in a sample of 3966 adolescents and emerging adults, and 2014 older adults. Using latent profile analysis, Barton and Miller (2015) identified subgroups of participants that were homogeneous in terms of the level of daily spiritual experiences and the level of positive psychology traits (namely forgiveness, gratitude, optimism, grit, and meaning). Notably, the established profiles differed with regard to depression and substance abuse, which may suggest that various combinations of positive psychological characteristics have a unique influence on outcome variables.

Given that positive and negative spiritual coping, as well as various aspects of moral virtues, are theoretically related but distinct from each other (Charzyńska 2015; Cook 2004; Pargament 1997; Peterson and Seligman 2004), and taking into account that various dimensions of spirituality may lead to different alcohol treatment outcomes (Krentzman et al., 2017; Robinson et al. 2011; Webb et al. 2006), there is a need to study their constellations instead of “atomizing” the spiritual sphere by studying them in isolation. Hence, the purpose of this study is twofold: (1) to identify distinct profiles of alcohol-dependent persons with similar patterns of spiritual coping, forgiveness, and gratitude; (2) to examine whether the patients who belong to different profiles would differ in terms of completion rates for an alcohol addiction treatment program. To answer these questions, a person-centered approach was applied (Vermunt and Magidson 2005). This approach, in contrast to a variable-centered approach, assumes that the population is heterogeneous in terms of study variables (indicators), and the goal of the analysis is to distinguish population subgroups of people who are similar to each other (i.e., are internally homogeneous), and who differ from people in other profiles (Collins and Lanza 2010).

The study was exploratory in nature, and thus, no specific hypotheses on the exact number of profiles were formulated. Nevertheless, it was expected that at least three profiles would be derived: one made up of people with high levels of positive spiritual coping, forgiveness, and gratitude along with a low level of negative spiritual coping; another profile with a pattern of indicators opposite to the first profile; and one profile with high levels of all indicators (i.e., both positive and negative dimensions of spirituality). With regard to treatment outcomes, it was hypothesized that members of the profile with high levels of positive spiritual coping, forgiveness, and gratitude, and low levels of negative spiritual coping would be more likely to complete a basic alcohol addiction program compared to members of the other profiles (Giordano et al. 2015; Robinson et al. 2011; Webb et al. 2006). Furthermore, it was expected that patients with an opposite pattern of spiritual coping, forgiveness, and gratitude compared to the first profile would be most likely to drop out from alcohol addiction treatment. It was also assumed that members of the profile with high levels of all dimensions of spirituality would be less likely than members of the first profile but more likely than members of the second profile to complete alcohol addiction therapy.

## Method

### Participants

The sample included 358 Polish participants receiving short-term outpatient treatment in alcohol dependence treatment centers in southern Poland. Fifteen participants returned empty questionnaires (a participation rate of 96%); thus, they were excluded from the study. Moreover, because some of the variables of interest (namely positive and negative religious coping, and feeling forgiven by God) were directly related to religiousness, the data from 20 patients (5.8%) who declared that they did not believe in God/a higher being were also excluded from further analysis.

The final sample consisted of 323 participants. Table 1 presents the sociodemographic and alcohol-related characteristics of the sample. The majority of participants were male, Roman Catholic, graduates of elementary or vocational schools, unemployed or pensioners, and in intimate relationships.

**Table 1:** Sample Characteristics  (*N* = 323)

Variable	*n*	%
Gender		
Male	230	71.2
Female	93	28.8
Age in years (*M* ± SD)	42.94	10.39
Denomination		
Roman Catholic	280	86.7
Evangelical	3	0.9
Greek Catholic	1	0.3
Not affiliated with any particular denomination	39	12.1
Education		
Elementary	54	16.7
Vocational	148	45.8
Secondary	96	29.7
Higher	24	7.5
N/A	1	0.3
Professional status		
Employed	135	41.8
Unemployed	147	45.5
Pensioner	40	12.4
N/A	1	0.3
Marital status		
Single	158	48.9
In relationship	165	51.1
Duration of alcohol dependence in years (*M* ± SD)	14.14	8.57
Number of previous alcohol addiction therapies (*M* ± SD)	1.33	1.90
Attendance of AA meetings		
No	310	96.0
Yes	13	4.0

*N* = 323

*M* mean, *SD* standard deviation

## Measures

### Spiritual Coping

The Spiritual Coping Questionnaire (SCQ; Charzyńska 2015) was used to assess patients’ spiritual coping. Participants were presented with 32 items measuring spiritual coping in various domains and were asked to indicate what they were thinking and doing in the past four weeks when dealing with their alcohol problems. The SCQ items are divided into seven subscales, constituting two main scales: positive spiritual coping and negative spiritual coping. Positive spiritual coping is made up of four subscale domains: personal (four items; e.g., “I was trying to find sense in what happened”), social (six items, e.g., “I was compassionate toward other people’s pain”), environmental (five items, e.g., “I was seeking closeness to nature”), and religious (six items, e.g., “In my relations with God/a higher being, I was searching for strength to live”). Negative spiritual coping consists of three subscale domains: personal (four items, e.g., “I was convincing myself that my life had no sense”), social (four items, e.g., “I was convincing myself that other people were full of evil”), and religious (three items, e.g., “I was blaming God/a higher being for what happened in my life”). The participants answered using a 5-point Likert scale (1 = “very inaccurately,” 2 = “rather inaccurately,” 3 = “neither inaccurately nor accurately,” 4 = “rather accurately,” and 5 = “very accurately”). The scores for each of the seven subscales were calculated by averaging the responses to particular items. Cronbach’s alpha coefficients for the subscales of the SCQ ranged from 0.71 (negative religious coping) to 0.95 (positive religious coping).

### Forgiveness

The Forgiveness Scale (Charzyńska and Heszen 2013), which is a Polish adaptation of indices of forgiveness prepared by Toussaint et al. (2001), was used to measure forgiveness. The scale encompasses three aspects of forgiveness: self-forgiveness (two items; e.g., “I find it hard to forgive myself for some of the things I have done wrong”), forgiveness of others (five items; e.g., “I have forgiven those who have hurt me”), and feeling forgiven by God (two items; e.g., “I know that God forgives me”). The response format for the Forgiveness Scale depends on the item formulation. The participants respond using a 5-point Likert scale ranging from 1 (“strongly agree” or “never”) to 5 (“strongly disagree” or “very often”). Five items had to be recoded because they measured difficulty with self-forgiveness or inability to forgive others. The indices of particular aspects of forgiveness were calculated by averaging the responses to particular items. In the current study, self-forgiveness, forgiveness of others, and feeling forgiven by God yielded an internal consistency estimate (Cronbach’s alpha coefficient) of 0.76, 0.75, and 0.90, respectively.

### Gratitude

The trait gratitude was measured with the Gratitude Questionnaire (GQ-6; McCullough et al. 2002; Polish adaptation by Kossakowska and Kwiatek 2014). The GQ-6 is made up of six items (e.g., “If I had to list everything that I felt grateful for, it would be a very long list”). The responses are given using a 7-point Likert scale (1 = “strongly disagree,” 2 = “disagree,” 3 = “slightly disagree,” 4 = “neutral,” 5 = “slightly agree,” 6 = “agree,” and 7 = “strongly agree”). Two items were reverse scored because they measured difficulty expressing or feeling gratitude. The level of gratitude was calculated by summing up all the responses. In this study, Cronbach’s alpha coefficient for the GQ-6 was 0.71.

## Procedure

The research procedures were carried out in accordance with the Declaration of Helsinki and were approved by the Institutional Research Ethics Committee at the University of Silesia in Katowice, Poland. The study was carried out at the day care wards of 11 public alcohol dependence treatment centers in southern Poland. The inclusion criteria were as follows: (1) diagnosis of alcohol dependence in accordance with the 10th revision of the International Classification of Diseases and Related Health Problems (ICD-10; World Health Organization 2010); (2) currently in an alcohol addiction treatment program; (3) aged 18 years or older; and (4) provided written informed consent.

Patients participated in group outpatient therapy on weekdays for six to eight weeks, depending on the center. They also attended individual therapy sessions, during which the patient’s current problems were discussed and individual plans of abstinence were prepared. All the centers follow a similar abstinence-focused protocol, integrating elements of the Minnesota model, social learning theory, cognitive-behavioral therapy, motivational enhancement therapy, existential-humanistic therapy, and the experience of the AA movement. Patients are also encouraged by the staff to attend AA meetings. The therapy program covers some topics related to spirituality and moral virtues but these are not a primary focus of the program. Accordingly, none of the treatment centers introduce controlled interventions targeted at enhancing patients’ spirituality or moral virtues.

The baseline measurement was carried out during the first week of the treatment by trained research assistants. The lists of patients who either completed or dropped out from the treatment program were provided by the therapists once a fortnight. All the personal details of the patients were anonymized using pseudonyms.

### Statistical Analysis

Before verification of the hypotheses, Little’s Missing Completely at Random test was performed to examine whether the data were missing completely at random (Little and Rubin 1987). The expectation–maximization (EM) algorithm (Dempster et al. 1977) was used to impute missing data. In the next step of analysis, means, standard deviations, and paired comparisons, along with correlation coefficients for spiritual coping, forgiveness, gratitude, and therapy completion, were calculated.

To test the hypotheses, a latent profile analysis (LPA), which is one of the methods of the person-centered approach, was conducted. LPA is an extension of latent class analysis (LCA) that uses continuous variables as indicators of profile membership (Vermunt and Magidson 2005). LPA enables the grouping of individuals in relatively homogeneous subpopulations, presenting qualitatively and quantitatively distinct patterns on a set of indicators. It has been demonstrated that LPA outperforms traditional clustering procedures (e.g., K-means or hierarchical clustering; Magidson and Vermunt 2002; Meyer et al. 2013).

In the first step of LPA, models containing one to seven profiles were examined and compared using the following information criteria (Nylund et al. 2007; Tein et al. 2013): Bayesian information criterion (BIC), consistent Akaike information criterion (CAIC), and sample-size adjusted BIC (SABIC). Lower BIC, CAIC, and SABIC values indicate better model fit. To estimate the precision with which cases are assigned to profiles, the entropy value was calculated; higher entropy values (i.e., values closer to one) represent better latent profile separation (Magidson and Vermunt 2002). In addition, to provide stable and meaningful latent profile solutions several other criteria were taken into account: model parsimony (in favor of a less complex model), latent profile proportions (the smallest profile could not be made up of less than 5% of the total sample), and substantive interpretability of the profiles (Collins and Lanza 2010).

Once the best solution was determined, the participants were classified into latent profiles on the basis of their probability scores. Finally, using a Wald test, the probability scores were related to an outcome variable, that is, completion of therapy (Vermunt 2010). The calculations were performed in Latent GOLD 5.1 (submodule called Step 3 included; Vermunt and Magidson 2005) and IBM SPSS Statistics version 25 (IBM Corp. 2017).

## Results

### Missing Data

The percentage of missing values was 0.88%. The results of the Little’s test (*χ*^2^(150) = 161.68; *p* > 0.05) showed that the data followed the pattern of being completely at random. Thus, in the next step a single imputation by EM algorithm (Dempster et al. 1977) was performed using the Missing Value Analysis Module in IBM SPSS version 25.0 (IBM Corp. 2017).

### Preliminary Analysis

Table 2 presents descriptive statistics along with bivariate correlations for all study variables. In most cases, positive spiritual coping was positively associated with forgiveness of others, feeling forgiven by God, and gratitude, but not with self-forgiveness; the inverse correlations were found between negative spiritual coping and all moral virtues. Moreover, gratitude was positively related to all aspects of forgiveness. All the correlations between indicators were either weak or moderate, which suggested that they measured different psychological constructs and thus justified employing LPA. Positive spiritual coping in the personal domain turned out to be the only significant correlate of therapy completion (*r* = 0.16, *p* = 0.005).

**Table 2:** Descriptive statistics and correlations between study variables

Variables	*M*	SD	Range	*α*	1	2	3	4	5	6	7	8	9	10	11	12
1. Positive personal coping	3.62	0.86	1–5	.80	1											
2. Positive social coping	3.85	0.82	1–5	.87	.48***	1										
3. Positive environmental coping	3.20	1.16	1–5	.91	.40***	.48***	1									
4. Positive religious coping	2.97	1.27	1–5	.95	.39***	.39***	.42***	1								
5. Negative personal coping	2.66	1.24	1–5	.77	− .14*	− .14*	− .13*	− .06	1							
6. Negative social coping	2.12	0.96	1–5	.78	− .12*	− .10	.02	.11*	.47***	1						
7. Negative religious coping	2.16	1.11	1–5	.71	− .11*	− .05	.01	.15**	.46***	.42***	1					
8. Self-forgiveness	2.37	0.94	1–5	.76	− .03	− .03	.07	− .04	− .32***	− .21***	− .25***	1				
9. Forgiveness of others	3.25	0.77	1–5	.75	.22***	25***	.22***	.05	− .25***	− .35***	− .25***	.13*	1			
10. Feeling forgiven by God	3.53	1.12	1–5	.90	.23***	.24***	.22***	.53***	− .19**	− .07	− .05	.12*	.18**	1		
11. Gratitude	29.34	6.17	6–42	.71	.32***	.24***	.26***	.26***	− .23***	− .14*	− .17**	.16**	.23***	.26***	1	
12. Completion of therapy	–	–	–	–	.16**	.08	.05	.05	− .07	− .06	− .06	.01	.03	.04	.03	1

Completion of therapy was dummy-coded (0 = dropouts, 1 = completers). *N* = 323

*M*, mean; *SD*, standard deviation; *α*, Cronbach’s alpha coefficient

*p < .05; **p < .01; ***p < .001

### Hypothesis 1: LPA Profiles

Table 3 presents the comparison of LPA models for spiritual coping, forgiveness, and gratitude. The lowest BIC and CAIC values were noted for the five-profile solution. Although the SABIC value was the lowest for the seven-profile solution, this model was rejected due to a very low frequency for the smallest profile (3.8%), suggesting the instability of this solution. This decision was also supported by the investigation of the shape of the profiles: two pairs of the profiles in the seven-profile solution differed from each other just slightly in terms of profile conditional response means, whereas no substantial differences in the shapes of these profiles were noted. After rejecting the seven-profile solution, the five-profile and the six-profile solutions were investigated more closely. The BIC, CAIC, and SABIC values were lower for the five-profile solution than for the six-profile solution. Accordingly, the former solution was identified as best fitting the data, and the latter was rejected.

**Table 3:** Summary comparison of LPA models

	Model						
	1-Profile	2-Profile	3-Profile	4-Profile	5-Profile	6-Profile	7-Profile
LL	− 5653.22	− 5435.85	− 5273.90	− 5199.44	− **5043.68**	− 5023.93	− 4935.47
BIC	11,433.55	11,131.69	10,940.69	10,924.65	**10,774.91**	10,839.39	10,795.36
CAIC	11,455.55	11,176.69	11,008.69	11,015.65	**10,893.91**	10,976.39	10,955.36
SABIC	11,363.77	10,988.95	10,725.00	10,636.00	**10,397.46**	10,404.85	10,287.86
Entropy	1.0	.80	.87	.87	**.87**	.87	.89
Smallest profile (%)	–	36.5	15.4	6.8	**11.6**	6.3	3.8

Bold values represent a best-fitting model. *N* = 323

*LL* model log-likelihood, *BIC* Bayesian information criterion, *CAIC* consistent Akaike information criterion, *SABIC* sample-size adjusted BIC

**Fig. 1:**
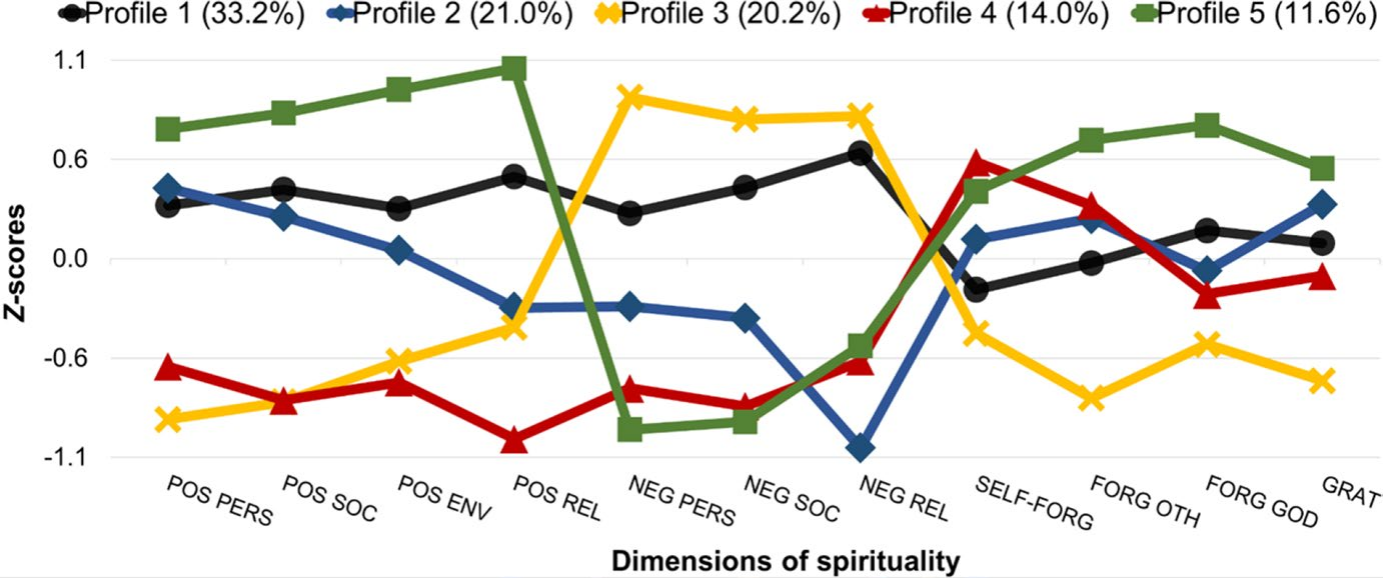
Five profiles of patients with different levels of spiritual dimensions indicated by LPA. For presentation purposes, mean values of all indicators (i.e., dimensions of spirituality) were converted to standardized values (i.e., *z*-scores). Profile 1 = moderately positive and negative dimensions of spirituality; Profile 2 = moderately positive dimensions of spirituality; Profile 3 = predominantly negative dimensions of spirituality. Profile 4 = mixed dimensions of spirituality with the lowest level of positive religious coping. Profile 5 = highly positive dimensions of spirituality. POS = positive; NEG = negative; PERS = personal; SOC = social; ENV = environmental; REL = religious; SELF-FORG = self-forgiveness; FORG OTH = forgiveness of others; FORG GOD = feeling forgiven by God; GRAT = gratitude. *N* = 323

Table 4 and Fig. 1 present five profiles identified by LPA. Almost one third of the patients belonged to Profile 1, which was labeled “both moderately positive and negative dimensions of spirituality.” These participants had moderate levels of all indicators: both positive (i.e., positive spiritual coping, forgiveness, gratitude) and negative ones (i.e., negative spiritual coping). Profile 2 (21.0%; “moderately positive dimensions of spirituality”) was composed of patients with moderate levels of positive spiritual coping and moral values, and a relatively low level of negative spiritual coping. Profile 3 (20.2%; “predominantly negative dimensions of spirituality”) grouped patients with low levels of positive spiritual coping (except for positive religious coping), forgiveness, and gratitude, and a high level of negative spiritual coping in all domains. Profile 4 (14.0%; “mixed dimensions of spirituality with the lowest positive religious coping”) was made up of patients with a low level of positive spiritual coping, especially positive religious coping, a relatively high level of self-forgiveness, moderate levels of forgiveness of others, feeling forgiven by God, and gratitude, and a low level of negative spiritual coping. The least numerous Profile 5 (11.6%; “highly positive dimensions of spirituality”) consisted of persons with high levels of positive spiritual coping and all moral virtues accompanied by a low level of negative spiritual coping.

**Table 4:** Conditional response means, standard deviations, and paired comparisons for the five-profile solution

Variable	Range	Profile 1 (33.2%)	Profile 2 (21.0%)	Profile 3 (20.2%)	Profile 4 (14.0%)	Profile 5 (11.6%)
		M (SD)	M (SD)	M (SD)	M (SD)	M (SD)
Positive personal coping	1–5	3.87 (0.75)^a^	3.96 (0.64)^a^	2.85 (1.03)^b^	3.10 (1.15)^b^	4.24 (0.56)^c^
Positive social coping	1–5	4.16 (0.81)^a^	4.04 (0.72)^a^	3.20 (0.87)^b^	3.21 (0.94)^b^	4.50 (0.43)^c^
Positive environmental coping	1–5	3.52 (1.12)^a^	3.25 (1.18)^a^	2.54 (1.25)^b^	2.41 (1.24)^b^	4.28 (0.64)^c^
Positive religious coping	1–5	3.55 (1.02)^a^	2.63 (1.52)^b^	2.49 (1.18)^b^	1.70 (0.98)^c^	4.31 (0.73)^d^
Negative personal coping	1–5	2.97 (1.34)^a^	2.34 (1.06)^b^	3.77 (1.26)^c^	1.77 (0.91)^d^	1.49 (0.54)^d^
Negative social coping	1–5	2.50 (1.02)^a^	1.81 (0.76)^b^	2.86 (1.00)^c^	1.34 (0.48)^d^	1.26 (0.34)^d^
Negative religious coping	1–5	2.78 (1.04)^a^	1.10 (0.09)^b^	3.02 (1.16)^a^	1.52 (0.61)^c^	1.62 (0.54)^c^
Self-forgiveness	1–5	2.21 (0.90)^ab^	2.47 (1.23)^a^	1.98 (0.83)^b^	2.90 (1.05)^c^	2.72 (0.93)^c^
Forgiveness of others	1–5	3.23 (0.80)^a^	3.42 (0.78)^a^	2.66 (0.84)^b^	3.48 (0.63)^a^	3.76 (0.59)^c^
Feeling forgiven by God	1–5	3.70 (1.04)^a^	3.45 (1.46)^ab^	3.00 (1.27)^b^	3.31 (1.02)^b^	4.35 (0.76)^c^
Gratitude	7–42	29.87 (5.23)^a^	31.20 (6.76)^ac^	25.19 (7.37)^b^	28.75 (6.57)^a^	32.43 (5.87)^c^

Means within rows with differing superscripts differ significantly at the *p* < .05 level. Profile 1 = both moderately positive and negative dimensions of spirituality; Profile 2 = moderately positive dimensions of spirituality; Profile 3 = predominantly negative dimensions of spirituality; Profile 4 = mixed dimensions of spirituality with the lowest level of positive religious coping; Profile 5 = highly positive dimensions of spirituality. *N* = 323

*M* mean, *SD* standard deviation

### Hypothesis 2: Relationship Between LPA Profiles and Therapy Completion

From 323 participants of this study, an alcohol addiction therapy program was completed by 170 persons (52.6%), including 117 men (50.9% of all men) and 53 women (57.0% of all women). The Wald test showed that completion of therapy was significantly related to the latent profile membership (Wald = 14.15; *p* = 0.007). The completion rates for each profile were as follows: 0.538 for Profile 1 (“both moderately positive and negative dimensions of spirituality”), 0.584 for Profile 2 (“moderately positive dimensions of spirituality”), 0.482 for Profile 3 (“predominantly negative dimensions of spirituality”), 0.301 for Profile 4 (“mixed dimensions of spirituality with the lowest positive religious coping”), and 0.738 for Profile 5 (“highly positive dimensions of spirituality”). Patients in Profile 4 were less likely to complete therapy compared to patients in Profile 1 (Wald = 5.83; *p* = 0.016), Profile 2 (Wald = 7.07; *p* = 0.0079), and Profile 5 (Wald = 12.75; *p* < 0.001). The difference between the members of Profile 4 and Profile 3 was also noted but it did not achieve a statistically significant level of *p* < 0.05 (Wald = 2.99; *p* = 0.084). Moreover, patients in Profile 5 completed therapy more often not only than patients in Profile 4, but also than patients in Profile 1 (Wald = 3.89; *p* = 0.049) and Profile 3 (Wald = 5.41; *p* = 0.030).

## Discussion

### Latent Profile Membership and Its Association with Treatment Completion

The sample of patients of alcohol addiction therapy turned out to be heterogeneous in terms of spiritual coping, forgiveness, and gratitude, which is indicated by five established profiles with different qualitative and quantitative characteristics. Importantly, persons who belonged to different profiles also differed in terms of the rates of treatment completion. Hence, not only do present findings lend strong support to the multidimensional nature of spirituality, but they also, and primarily, indicate that various baseline dimensions of spirituality impact the completion of alcohol addiction therapy to different extents.

As expected, LPA identified the profile with high levels of positive spiritual coping, forgiveness, and gratitude, and a low level of negative spiritual coping (Profile 5). This profile had the greatest probability of completing treatment (0.738), which confirms, consistent with the previous studies (Chen 2017; Laudet and White 2008; Medlock et al. 2017; Pardini et al. 2000; Webb et al. 2006), that spiritual coping, forgiveness, and gratitude build personal and social resources that support recovery from alcohol dependence.

The profile with moderate levels of both positive and negative spiritual variables (Profile 1) turned out to be the most numerous. Approximately one third of persons entering alcohol addiction therapy resorted to both positive and negative dimensions of spirituality. For persons addicted to alcohol, the time periods immediately before and after admission into treatment often bring acute stress as the individual has to deal with the consequences of heavy drinking and psychosocial problems (Puffer et al. 2012). As a consequence, the time periods around early recovery from alcohol dependence seem to stimulate the patients’ use of spirituality to cope with stress, in both positive and negative ways.

In this context, it is important to take a closer look at the results of comparison of Profiles 1 and 5. According to the findings, Profile 1 (“both moderately positive and negative dimensions of spirituality”) had a lower probability of completing alcohol addiction therapy than Profile 5 (“highly positive dimensions of spirituality”). This means that resorting to negative spiritual coping when facing stress—even if positive spiritual resources are used simultaneously—decreases the completion rates for alcohol addiction treatment. This is consistent with the results of previous studies, which noted the detrimental effects of negative religious coping on the outcomes of substance abuse treatment (Puffer et al. 2012; Robinson et al. 2011). The tendency to engage in maladaptive spiritual coping may be associated with a sense of hopelessness about one’s ability to change and other negative affective states that lead to increased alcohol cravings and thus heighten the risk for relapse (Giordano et al. 2015; Medlock et al. 2017).

In accordance with expectations, LPA yielded a profile with relatively low levels of positive spiritual coping, forgiveness, and gratitude along with a high level of negative spiritual coping (Profile 3). However, quite surprisingly, members of another profile (Profile 4, “mixed dimensions of spirituality with the lowest positive religious coping”) turned out to have the highest probability of dropping out from therapy. This result suggests that a very low level of positive religious coping may significantly hamper completion of treatment, even if a person rarely uses negative spiritual coping and has mostly average levels of forgiveness and gratitude. Not resorting to religious sources when coping with alcohol dependence–related stress may limit patients’ chances to experience the numerous benefits of positive religious coping, such as providing a meaningful personal framework; enhancing self-efficacy, optimism, and hope; serving as a buffer against depression; or mitigating alcohol cravings (Giordano et al. 2015; Medlock et al. 2017).

It should be mentioned in this context that members of Profile 4 had a higher level of self-forgiveness compared to Profiles 1–3 (Table 4). A substantial number of studies have demonstrated positive relationships between self-forgiveness and treatment outcomes among alcohol-dependent persons (Robinson et al. 2011; Webb et al. 2006). Furthermore, there is robust evidence that self-forgiveness may be the most important aspect of forgiveness in the treatment of alcohol dependence (Webb et al. 2011). However, there is also some evidence that higher baseline self-forgiveness may be associated with higher probability of dropping out from drug and alcohol treatment programs (Deane et al. 2012). A high baseline level of self-forgiveness may in fact indicate the occurrence of the ego-defensive process called pseudo self-forgiveness which should be clearly differentiated from genuine self-forgiveness. Pseudo self-forgiveness takes place when a person does not accept personal responsibility for the wrongdoing or refuses to acknowledge that the offending behavior was wrong (Hall and Fincham 2005). The possibility of the occurrence of pseudo self-forgiveness among members of Profile 4 is supported by a low level of negative spiritual coping in a personal domain noted in this group, compared to members of Profiles 1–3. Overall, the combination of a very low baseline level of positive religious coping accompanied by a relatively high baseline level of self-forgiveness seems crucial to explain the lowest rates of treatment completion noted in this profile. Religious coping is sometimes used by persons with high levels of self-blame and self-resentment to mitigate negative emotions toward self (Wasserman et al. 2013). This mechanism does not seem to work for members of Profile 4 who may perceive using religious sources of coping as unnecessary. These patients are likely to maintain the illusion of self-reliance because of which they can deny loss of control over their alcohol use (Chen 2017). The topic of the relationships between self-forgiveness, pseudo self-forgiveness, and positive religious coping among alcohol-dependent persons needs further investigation.

### Clinical Implications

The results of the current study have some practical implications that are worth discussing. As noted, less than 12.0% of the sample (i.e., Profile 5) of 323 participants who declared a belief in God/a higher being presented a pattern of spiritual coping, aspects of forgiveness, and gratitude that was highly supportive of therapy completion. This finding indicates a need to routinely measure the initial levels of these variables during admission to alcohol addiction treatment to build a robust basis for subsequent practice. This is in accordance with the recommendations of many scholars and clinicians (Grim and Grim 2019; Hodge 2001) as well as with professional guidelines for therapists (e.g., Association for Spiritual, Ethical, and Religious Values in Counseling 2009) and codes of ethics (e.g., American Counseling Association 2014). However, although many mental health professionals have acknowledged the benefits of incorporating clients’ spirituality in treatment, it is still not a standard practice in therapy. In practice, usually little time and effort is devoted to addressing this topic during treatment (Cornish et al. 2012), even though many patients express the wish for incorporating their spiritual beliefs into the therapeutic enterprise (Dermatis et al. 2004). With regard to alcohol addiction treatment, therapists usually limit themselves to encouraging patients to attend AA meetings (Miller 2013). This reluctance to include spirituality in practice has various reasons, including insufficient knowledge, lack of experience, fears, or ethical concerns (Cornish et al. 2012). This is why specialized training courses and workshops concerning spirituality should be offered to alcohol addiction therapists. Professional training would help them to gain knowledge and competencies needed to take full advantage of patients’ spiritual resources and to prevent the risk of relapse related to the spiritual struggle faced by some patients.

When working with persons with alcohol dependence, individual treatment plans should expand on issues related to spiritual potential and deficits. Knowledge of the specific configuration of dimensions of spirituality for a given patient would make it possible to develop focused clinical interventions aimed at modifying specific spiritual variables, adjusted to the needs and expectations of the patient, and his or her current situation, in this way providing integrated holistic care (Medlock et al. 2017; Puffer et al. 2012; Grim and Grim 2019; Wong 2010). Several recommendations for modifying positive and negative religious coping have already been made in the literature and include, among others, combining spiritual issues with a cognitive-behavioral framework by encouraging positive religious coping thoughts and challenging negative ones, changing attachment styles and God-related images, or simply making more referrals to 12-step support groups (Grim and Grim 2019; Moriarty et al. 2006).

### Limitations

To the best of the author’s knowledge, this is the first study to explore the configurations of baseline dimensions of spirituality and their associations with the rates of successful completion of alcohol addiction treatment. Despite a number of strengths, it has some limitations in need of acknowledgment and consideration. One group of limitations pertains to sample characteristics. Although the sample was quite large and heterogeneous in terms of sociodemographic and alcohol-related variables, the study involved only those individuals who participated in an outpatient group therapy. Thus, the results should not be generalized to other contexts, such as inpatient treatment. Moreover, because of the religious character of some of the variables measured, only data from patients who declared a belief in God or another higher being were included in the analyses, which may limit the generalizability of the research findings to some extent. However, it should be noted that the rate of exclusion due to being non-religious was low (5.8%).

Another limitation stems from the measures used in the study. First, the indexes of self-forgiveness and feeling forgiven by God consisted of only two items, which could have made it difficult to capture intra- and interindividual variability of the scores. Nevertheless, these indexes turned out to be sufficient to observe the differences between the profiles; also, the reliability indexes for these measures were acceptable in this study. Second, gratitude was operationalized as a general disposition only. It is recommended that future studies include different aspects of gratitude (e.g., gratitude toward God, gratitude toward therapists, gratitude for sobriety) to determine how they are related to treatment completion.

In the current study, the completion of therapy was regarded as the only outcome of interest. No other clinically relevant variables (e.g., abstinence self-efficacy, alcohol cravings, quality of life) were taken into account. In addition, this study measured the completion rates for short-term therapy and did not monitor patients’ alcohol-related behaviors for a longer period of time. Thus, future studies should apply longer follow-up intervals to examine whether baseline configurations of spiritual coping, forgiveness, and gratitude are important for long-term abstinence.

Finally, when interpreting the results, the specific cultural context in which this study was conducted should be taken into account. Poland is a religiously homogeneous country; more than 90% of Poles declare themselves to be religious, with most identifying as Roman Catholic (Central Statistical Office 2018; Zarzycka 2009). Thus, for most Poles, spirituality manifests in a traditional religious form. It has been noted that living in a religious culture may influence the importance of religious variables in the context of recovery from alcohol dependence (Webb et al. 2011). Polish people may be more likely to use religious resources when struggling with stressful situations—such as beginning alcohol addiction therapy—compared to persons living in more secular countries (Strobbe et al. 2013). Moreover, in some countries, a group of people who declare themselves to be spiritual but not religious has been growing substantially (Fuller and Parsons 2018). Further research is therefore required to examine the role of dimensions of spirituality in treatment completion in more spiritually and religiously diverse countries than Poland.

## Conclusions

The current study confirms the benefits of applying a person-centered approach for exploring the combinations of dimensions of spirituality among persons beginning alcohol addiction therapy. As shown, this approach equips researchers and practitioners with better knowledge and understanding of the specific role played by distinct patterns of baseline spiritual coping, aspects of forgiveness, and gratitude in the completion of alcohol addiction therapy. The study results point out the necessity of the assessment of multiple spiritual variables at the time of admission for alcohol addiction treatment. The findings also suggest the importance of considering patients’ spiritual capabilities and deficits when preparing the treatment protocol, individual plans, and tailored interventions. Last but not least, they underscore the importance of professional training for therapists in the area of spirituality in order to provide them with competencies needed to adequately identify patients’ spiritual coping strategies and moral virtues that decrease or increase the risk of dropping out from treatment.
